# Supplementary material for: A high-resolution mRNA expression time course of embryonic development in zebrafish
Source: eLife. 2017 Nov 16;6:e30860. doi: 10.7554/eLife.30860 (PMC5690287; doi:10.7554/eLife.30860)
Supplement: Supplementary file 6. [file elife-30860-supp6.zip › biolayout-clusters-files/Cluster115-genes.html]

Cluster115


# Cluster115: Genes

| Ensembl ID | Gene Name | Chr | Start | End | Biotype |
| --- | --- | --- | --- | --- | --- |
| ENSDARG00000087369 | cep162 | 16 | 37941723 | 37994448 | protein\_coding |
| ENSDARG00000063167 | chkb | 18 | 8362146 | 8380927 | protein\_coding |
| ENSDARG00000069742 | cul5a | 15 | 17208953 | 17233913 | protein\_coding |
| ENSDARG00000035924 | dync1li2 | 7 | 44335731 | 44365685 | protein\_coding |
| ENSDARG00000004843 | foxp1a | 23 | 10703039 | 10787730 | protein\_coding |
| ENSDARG00000011948 | insra | 2 | 37562494 | 37650439 | protein\_coding |
| ENSDARG00000060348 | map3k3 | 3 | 31681660 | 31714102 | protein\_coding |
| ENSDARG00000057513 | mdm4 | 11 | 22637980 | 22663624 | protein\_coding |
| ENSDARG00000086317 | si:ch211-67e16.4 | 9 | 14052183 | 14070084 | protein\_coding |
| ENSDARG00000056549 | vps26a | 13 | 22927818 | 22936931 | protein\_coding |
| ENSDARG00000078571 | wdr6 | 8 | 26210921 | 26221366 | protein\_coding |
